# Iron and Cadmium Entry Into Renal Mitochondria: Physiological and Toxicological Implications

**DOI:** 10.3389/fcell.2020.00848

**Published:** 2020-09-02

**Authors:** Frank Thévenod, Wing-Kee Lee, Michael D. Garrick

**Affiliations:** ^1^Faculty of Health, Centre for Biomedical Education and Research, Institute of Physiology, Pathophysiology and Toxicology, Witten/Herdecke University, Witten, Germany; ^2^Department of Biochemistry, Jacobs School of Medicine and Biomedical Sciences, University at Buffalo, Buffalo, NY, United States

**Keywords:** reactive oxygen species, divalent metal transporter 1, ionic mimicry, manganese, copper, nephrotoxicity, acute kidney injury, chronic kidney disease

## Abstract

Regulation of body fluid homeostasis is a major renal function, occurring largely through epithelial solute transport in various nephron segments driven by Na^+^/K^+^-ATPase activity. Energy demands are greatest in the proximal tubule and thick ascending limb where mitochondrial ATP production occurs through oxidative phosphorylation. Mitochondria contain 20–80% of the cell’s iron, copper, and manganese that are imported for their redox properties, primarily for electron transport. Redox reactions, however, also lead to reactive, toxic compounds, hence careful control of redox-active metal import into mitochondria is necessary. Current dogma claims the outer mitochondrial membrane (OMM) is freely permeable to metal ions, while the inner mitochondrial membrane (IMM) is selectively permeable. Yet we recently showed iron and manganese import at the OMM involves divalent metal transporter 1 (DMT1), an H^+^-coupled metal ion transporter. Thus, iron import is not only regulated by IMM mitoferrins, but also depends on the OMM to intermembrane space H^+^ gradient. We discuss how these mitochondrial transport processes contribute to renal injury in systemic (e.g., hemochromatosis) and local (e.g., hemoglobinuria) iron overload. Furthermore, the environmental toxicant cadmium selectively damages kidney mitochondria by “ionic mimicry” utilizing iron and calcium transporters, such as OMM DMT1 or IMM calcium uniporter, and by disrupting the electron transport chain. Consequently, unraveling mitochondrial metal ion transport may help develop new strategies to prevent kidney injury induced by metals.

## Introduction

Together with the lungs and intestines, the kidneys maintain mammalian body fluid homeostasis by excreting most metabolic end-products and controlling the concentration of most constituents. Details of kidney morphology, structure of the functional unit, the nephron, and physiology are found in handbooks of renal physiology (Windhager, 1992; Brenner, 2008; Alpern et al., 2013). To prevent critical losses and ensuing deficiencies, nephrons, which form a tubular system, actively reabsorb essential nutrients and electrolytes from primary fluid as well as actively secrete metabolic wastes since the rate of waste production exceeds their rate of glomerular filtration. Transport of solutes and water across tubule membranes occurs via passive transport (diffusive flux through channels and carrier-mediated facilitated diffusion) and active transport (primary active pumps/ATPases, secondary or tertiary active electrochemical potential-driven transporters). Active transport requires energy from adenosine triphosphate (ATP) for activation of basolateral Na^+^/K^+^-ATPases to maintain active reabsorption and secretion processes.

## Nephron Transport and Metabolism: Focusing on Energy

Among other functions, relevant to this review is the role of the proximal tubule (PT) in taking up filtered proteins and peptides via an apical multi-ligand receptor complex, megalin:cubilin:amnionless (Christensen and Birn, 2002), including metalloproteins, e.g., transferrin (Tf), an iron [Fe (III)] binding protein, and metallothionein (MT), a cadmium [Cd (II)] binding protein. The loop of Henle (LOH) establishes the medullary hyperosmotic interstitium that is required for reabsorption of water in the collecting duct (CD), to generate small volumes of concentrated urine (“antidiuresis”), thus preserving water for the body. From the viewpoint of energetics, the “motor” of the concentration gradient in the medulla is active NaCl reabsorption into the interstitium in the thick ascending limb of the LOH (via apical Na^+^/K^+^/2Cl^–^ cotransport mediated by SLC12A1, basolateral Na^+^/K^+^-ATPase and Cl^–^ channel ClC-Kb/K2), and resulting in hypo-osmotic tubular fluid entering the distal tubule (DT) (Windhager, 1992; Brenner, 2008; Alpern et al., 2013). The DT reabsorbs divalent metal ions from the hypo-osmotic fluid, including Fe (II) via the divalent metal transporter (DMT1/SLC11A2), and the luminal fluid is further ion depleted by active NaCl symport via SLC12A3. Finally, the CD fine-tunes urine composition through hormonal regulation of principal (light) CD cells via aldosterone and antidiuretic hormone by transient, regulated incorporation of epithelial Na^+^ (ENaC) and aquaporin-2 (AQP2) water channels, respectively, into the apical membrane.

To drive these transport processes, Na^+^/K^+^-ATPase is highly expressed in the basolateral membrane of renal tubule cells (Katz, 1982). The Na^+^/K^+^-ATPase activity profile along the nephron shows highest activity in the thick ascending limb of the LOH and DT, followed by the PT, whereas Na^+^/K^+^-ATPase is present in lesser amounts in other segments (Katz et al., 1979). These differences are paralleled by surface area distribution of the basolateral membrane (e.g., via infolded plasma membranes), the mitochondrial density, and IMM surface (Pfaller, 1982; reviewed in Guder and Ross, 1984), indicating a functional relation between ATP-forming and ATP-using structures.

Two relevant pathways of ATP production are glycolysis versus mitochondrial respiration via fatty acid oxidation (FAO) plus oxidative phosphorylation (OXPHOS), with the latter predominant in the PT and LOH whereas the former is more prominent in the distal nephron. All nephron segments except the PT, utilize glycolysis. Glycolysis can be anaerobic or aerobic. With oxygen, cytosolic glycolysis drives shuttling of pyruvate across the IMM, to consumption in the tricarboxylic acid (TCA) cycle in the mitochondrial matrix (Mccommis and Finck, 2015). The TCA cycle does not directly produce much ATP, but NADH and FADH_2_ feed electrons into the electron transport chain (ETC) (see section “Electron Transport Chain”) prior to OXPHOS to drive ATP production (Fernie et al., 2004). In contrast, gluconeogenesis predominantly takes place in PT and CD (reviewed in Schmidt and Guder, 1976; Guder and Ross, 1984; Wirthensohn and Guder, 1986). Notably, acute kidney injury (AKI) induced by hypoxia or ischemia displays impaired energetics in highly metabolically active nephron segments, such as PT and thick ascending LOH, and is associated with mitochondrial dysfunction and ATP depletion (Basile et al., 2012) (see sections “Fe Overload and Renal Injury” and “Fe and Mitochondrial Damage”). Hence, mitochondria are of crucial importance for renal function by supplying ATP that is essential for Na^+^/K^+^-ATPase activity.

## Mitochondrial Functions: Determinants of Life and Death

### Electron Transport Chain

The ETC comprises five multimeric complexes (CI–CV) localized in the IMM. Electrons are shuttled from the multivalent metal core of one complex to the next via redox reactions, aided by ubiquinone and cytochrome c (cytC) on either side of CIII, and generating energy for transfer of protons from the matrix to the IMS. The electrons ultimately transfer to the final electron acceptor, oxygen, to form H_2_O (Letts and Sazanov, 2017). Protons flow back through complex V, the F_1_-F_0_ ATP synthase, using the energy stored in the two components of the matrix-directed proton-motive force, a pH differential and an electrical membrane potential (Δψm), a process called chemiosmosis, to form ATP. Release of the stored energy initiates two rotary motors: the ring of c subunits in F_0_ (around subunit a), along with subunits γ, δ and ε in F_1_, to which F_0_ is attached. Protons pass F_0_ via subunit a to the c-ring (Wittig and Schagger, 2008). Rotation of subunit γ within the F_1_ α3β3 hexamer provides energy for ATP synthesis. This process is called “rotary catalysis” (reviewed in Jonckheere et al., 2012). During proton translocation, a steep potential gradient is generated that exerts a force on a deprotonated glutamate, resulting in net directional rotation, converted into chemical energy (Klusch et al., 2017). This energy is stored in phospho-anhydride bonds in the ATP molecule, and then liberated in exergonic hydrolysis reactions by cellular ATPases to power energy-dependent cellular processes. A lateral chemical H^+^ gradient from complex IV of the ETC (lower local pH) to F_1_-F_0_ ATP synthase (higher local pH) resulting from the proton sink generated by proton transport through the F_1_-F_0_ ATP synthase may necessitate a modification to Peter Mitchell’s chemiosmotic model (Rieger et al., 2014). Further, some electrons “leak” back through the membrane as byproducts of single electron escape from ETC complexes, OXPHOS and matrix biochemical reactions, resulting in the production of noxious reactive oxygen species (ROS), usually superoxide anion (O_2_•^–^) or hydrogen peroxide (H_2_O_2_) (Munro and Treberg, 2017), which can be detoxified (see section “What to Do About/With So Many Radicals?”).

### Mitochondrial ROS Generation

**FIGURE 1:**
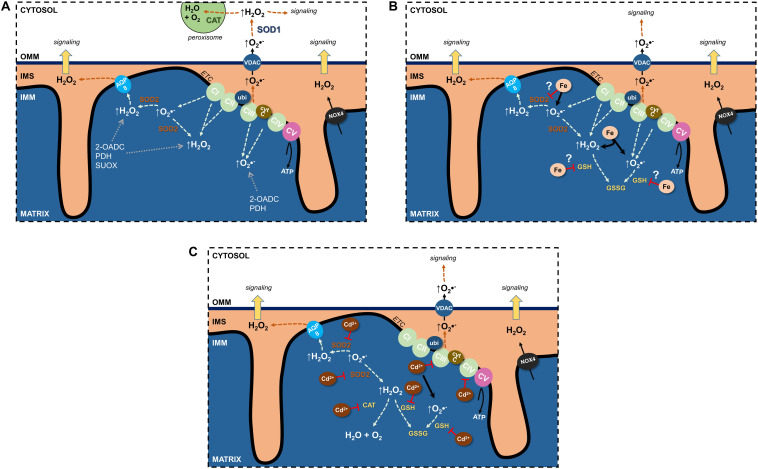
Effects of Fe and Cd on mitochondrial reactive oxygen species (ROS). **(A)** Sources of physiological ROS from mitochondria and their release into the cytosol. **(B)** Fe increase mitochondrial ROS formation and potentially inhibits antioxidants. **(C)** Cd inhibits the electron transport chain, binds GSH as well as decreases antioxidant enzyme activity to augment mitochondrial ROS generation. See text for further details. 2-OADC, 2-oxoacid dehydrogenase complexes; AQP, aquaporin; CAT, catalase; cytc, cytochrome c; ETC, electron transport chain; GSH, glutathione; IMS, intermembrane space; NOX4, NADPH oxidase 4; PDH, pyruvate dehydrogenase; SOD, superoxide dismutase; SUOX, sulfite oxidase; ubi, ubiquinone; VDAC, voltage-dependent anion channel.

Undoubtedly, the ETC is the major source of mitochondrial ROS (mtROS) (see section “Electron Transport Chain”) (Figure 1A). Through their flavin- and quinone-binding sites, CI and CIII are the largest ETC ROS generators (I > III > II >> IV), mostly superoxide, that are deposited into the matrix (Yan et al., 2020). CI and CII generate matrix H_2_O_2_, and CIII’s superoxide can also be found in the cristae lumen and IMS (Brand, 2016) where it traverses the OMM through the voltage dependent anion channel (VDAC) into the cytosol (Han et al., 2003) and can impact cellular signaling pathways following its conversion to H_2_O_2_.

Additional sources of mtROS stem from substrate metabolism and OXPHOS (Quinlan et al., 2014; Brand, 2016; Sies and Jones, 2020). In fact, the 2-oxoacid dehydrogenase complex (2-OADC) can generate approximately eight times the amount of ROS as CI (Quinlan et al., 2014)! Superoxide/H_2_O_2_ generated through these sources are deposited in the matrix. ROS can also be generated by more finely controlled activity of NADPH oxidases (NOX) located in cellular membranes and constitute a physiological signaling pathway (Bedard and Krause, 2007; Buvelot et al., 2019). NOX1–3 and NOX5 generate superoxide whereas NOX4 is constitutively active and generates H_2_O_2_ (Bedard and Krause, 2007; Nisimoto et al., 2014). NOX4 was localized to the IMM (Block et al., 2009) of renal PT HK-2 cells and was negatively regulated by ATP (Shanmugasundaram et al., 2017), suggesting OXPHOS activity uses ROS signals to regulate mitochondrial activity or signaling.

Notably, mitochondrial superoxide/H_2_O_2_ production can be modulated by both intra- and extramitochondrial cues, such as through proton leak back into the matrix, formation of super-complexes from one or more ETC complexes, or redox status (reviewed in Kuksal et al., 2017).

### Apoptosis Signaling

Apoptotic stimuli, involving calcium or ROS, induce the IMM to undergo mitochondrial permeability transition by triggering formation of a mitochondrial permeability transition pore (mPTP) (see also section “Cd and Kidney Mitochondrial Damage”) at contact sites between the IMM and OMM. The molecular composition of mPTP, once thought to contain OMM VDAC, IMM ANT, and matrix cyclophilin D, has remained elusive until now (reviewed in Baines and Gutierrez-Aguilar, 2018; Bonora and Pinton, 2019). Consequently, the IMM is no longer selective and there is a sudden increase in the permeability of the IMM to solutes up to 1500 Da, which also dissipates Δψ_*m*_. Uncontrolled flux of solutes and water into the matrix occurs, concluding with an increase in matrix volume due to osmotic pressure increase and matrix expansion. The IMM with its increased surface area disrupts the OMM culminating in the release of pro-apoptotic factors, like cytC or apoptosis inducing factor, from the IMS resulting in general mitochondrial dysfunction (Kroemer et al., 2007). Dissociation of cytC from cardiolipin is promoted by ROS resulting in its transfer from a tightly- to a loosely-bound pool (Petrosillo et al., 2003) [cytC is tightly bound to the IMM by electrostatic interactions (Ott et al., 2002)], triggering apoptosis (Brown and Borutaite, 2008).

## Metal Distribution in Mitochondria – To What Purpose?

An early study on ^56^manganese (Mn) flux in rats noted that metal uptake was enriched in liver, pancreas and kidney and found ∼40% of liver and ∼64% of renal uptake in mitochondria (Maynard and Cotzias, 1955; Sakurai et al., 1985). In the last decade, development of biophysical methods allows estimation not only of organelle Fe concentration *in situ* but also speciation (Dlouhy and Outten, 2013). Application to yeast (Holmes-Hampton et al., 2013) yielded quantitative results where the authors stated “cells contained ∼150 μM Fe, distributed primarily into … mitochondrial Fe.” Unfortunately, Lindahl’s group has not yet provided such analyses for renal cells. Mitochondria in human bronchial epithelial cells contain ∼50% of total Fe content (Ghio et al., 2013; Ghio et al., 2016). In yeast, published data (Pierrel et al., 2007) suggest that 20% of cellular copper (Cu) resides in mitochondria. The relative richness of the mitochondrial endowment with these transition metals is consistent with the high proportion of redox functions that remain encoded in the small circular DNA of mitochondria while apparently many other organellar functions migrated to nuclear DNA.

The redox ability of Fe, Cu, and Mn to transition from one to another species accounts for their unusual utility; redox properties can also generate free radicals leading mostly to toxic effects for the same group of metals. For Fe, the electron donor is Fe^2+^; the acceptor, Fe^3+^. Many of the roles dependent on this property have been recently reviewed (Dlouhy and Outten, 2013). Remarkably many of the processes take place largely in mitochondria. Heme, as a cofactor, begins and ends its synthesis there and participates via cytochromes in the ETC. Biosynthesis of Fe-sulfur cluster proteins also involves mitochondria and cytosol (Lill et al., 2012) and also results in multiple proteins that function in the organelle (Dlouhy and Outten, 2013). While non-heme Fe also participates in multiple proteins, sorting out which one has mitochondrial effects is challenging for most of them. Nevertheless, one critical role for Fe is participating in the reduction of ribo- to deoxyribonucleotides in mitochondria through nuclear gene *RRM2B* (Bourdon et al., 2007). The *Rrm2b^–/–^* mouse exhibited inactivated ribonucleotide reductase that appeared in all analyzed tissues, including renal.

A major function of Mn and Cu in mitochondria is their critical role in metal-dependent superoxide dismutases (SODs) to detoxify superoxide (Zelko et al., 2002; Baker et al., 2017). Superoxide generation and SODs are considered below in sections “ROS/RNS: Double-Edged Swords” and “SODs”. The IMS is the location where Cu-dependent cytC oxidase matures, with its function, denoted in its name, mitochondrial. Other elements of Cu metabolism in the organelle were recently reviewed (Baker et al., 2017).

## ROS/RNS: Double-Edged Swords

Essential to mammalian life, molecular or diatomic oxygen (O_2_) is a diradical with two unpaired electrons that participate in an unusual three-electron bond between each oxygen atom. The configuration of these unpaired electrons in each outer orbital shell makes O_2_ particularly susceptible to free radical formation (Halliwell, 1991). The acceptance of an electron results in an anionic form of O_2_, the superoxide anion O_2_•^–^, highly reactive yet short-lived (Hayyan et al., 2016). Other free radicals include the hydroxyl radical (•OH) and (hydrogen) peroxide [H_2_(O_2_•^–2^)]; henceforth, H_2_O_2_ (Munro and Treberg, 2017). Though H_2_O_2_ itself is not a free radical, •OH can be generated in the presence of redox metals (Fe, Cu) through the Fenton reaction. Moreover, superoxide can react with nitric oxide (NO•) to form peroxynitrite (ONOO^–^) and NO• with CO_2_ to yield nitrosoperoxycarbonate (ONOOCO_2_^–^). Similar to ROS, these reactive nitrogen species (RNS) are highly reactive and can oxidize thiols and nitrate proteins (Adams et al., 2015). Since RNS generation is directly dependent on superoxide (Valdez et al., 2018), focus will be placed on ROS.

### ROS as Physiological Signals

Reactive oxygen species have long been regarded as unwanted and dangerous molecules that elicit cellular damage and cause mutations but are commonly used by immune cells to kill pathogens via an oxidative burst. Mounting evidence point to physiological signaling by ROS (Shadel and Horvath, 2015; Sies and Jones, 2020). H_2_O_2_ is favored as a physiological signaling molecule due to its higher stability, selective reactivity with cysteine groups and longer half-life. Similar to other second messengers, ROS can be induced by physiological stimuli, such as cytokines or mechanical forces (Droge, 2002). ROS operate in signaling through chemical reactions, which lead to covalent modifications through redox-sensitive cysteines (Brigelius-Flohe and Flohe, 2011; Ray et al., 2012; Kalyanaraman et al., 2018). Indeed, H_2_O_2_ is involved in engaging a number of signaling cascades (reviewed in Veal et al., 2007; Holmstrom and Finkel, 2014), such as tyrosine-protein kinases Lyn and Syk (Patterson et al., 2015), as well as affecting transcription factors (Marinho et al., 2014).

These redox-regulated protein switches can only operate in signaling if they are coupled to reducing enzymatic systems, such as thioredoxins (TDRXs) or peroxiredoxins (PDRXs) (Cox et al., 2009) [and possibly glutathione (GSH)]. Termination of ROS signals occurs through antioxidant enzymes – and probably endogenous scavengers – which are likewise compartmentalized, with specific enzymes for mitochondria, cytosol, peroxisomes, and the extracellular space (Kaludercic et al., 2014).

Whilst large amounts of unregulated and non-compartmentalized ROS damage cells through oxidation of encountered proteins, lipids and nucleic acids, it has become increasingly apparent that ROS have physiological functions, such as intracellular signal transmission or as a feedback signal during monitoring of function. Key strategies to differentiate between sub-toxic and toxic ROS signals are to compartmentalize ROS, limit their amount, and control their diffusion capacity.

### What to Do About/With So Many Radicals?

Mitochondrial ROS have two discernable fates: neutralization through antioxidants or release into the extramitochondrial space (Figure 1). The constant threat of excessive ROS generation and subsequent potential damage resulted in several antioxidant defense systems in mitochondria (Mailloux, 2018). SOD2 and GSH (Mari et al., 2009) are major ROS-inactivating mechanisms and supplemented with additional antioxidant machinery including GSH peroxidases (GPXs) (Handy et al., 2009), TRXPs, PRDXs, thioredoxin 2 (TRX2), glutaredoxin 2 (GRX2), and NAD(P)H/NAD(P)^+^. Most antioxidants are located in the matrix, with the exception of PDRXs, which are located in the IMS to capture peroxides (such as H_2_O_2_) that are directed there or have escaped the matrix (Herrmann and Riemer, 2010; Sabharwal et al., 2013).

Reactive oxygen species from the IMS can affect both IMM and OMM proteins. Of particular consequence is the redox sensitive open probability of the abundant OMM VDAC that appears to be indirectly regulated by the OMM-anchored (2Fe-2S) mitoNEET or CDGSH iron sulfur domain 1 protein (Lipper et al., 2019). The phenomenon is central to superoxide-mediated apoptotic cytC release (Madesh and Hajnoczky, 2001).

#### SODs

Superoxide dismutases drive the reaction of two superoxide anions with two H^+^ ions to generate the more stable H_2_O_2_ and water (Fukai and Ushio-Fukai, 2011; Hayyan et al., 2016). Though H_2_O_2_ can form •OH radicals, it now becomes available as a substrate for several antioxidant enzyme systems, including catalase, GPXs and PDRXs, so that it can be more efficiently removed. In humans, there are three SOD isoforms: SOD1 classically viewed as cytosolic and dependent on copper and zinc; SOD2, mitochondria-targeted and dependent on manganese (Karnati et al., 2013); SOD3, secreted and localized in the extracellular space and dependent on copper and zinc (Fukai and Ushio-Fukai, 2011; Hayyan et al., 2016).

An interrelationship between SOD2 and the ETC prevents unwarranted ROS generation and potential damage (Figure 1A). Loss of SOD2 results in decreased activity of CI and CIII, thus adapting to the availability of antioxidant. This phenomenon also highlights the importance of the SOD2 defense system, with losses neither compensated through other existing antioxidant systems nor via upregulation of cytosolic SOD1 (reviewed in Holley et al., 2011). Indeed, no change in SOD1 was observed in a kidney specific SOD2 knockout mouse model, wherein kidney function was unchanged (Parajuli et al., 2011).

### ROS Neutralization by Imported Glutathione

Glutathione, the most abundant antioxidant, is synthesized through a two-enzyme reaction catalyzed by glutamate cysteine ligase and glutathione synthetase. It is a tripeptide nucleophile with thiol groups capable of accepting electrons in its reduced state (GSH), thereby becoming oxidized (GSSG), and rendering ROS to a lesser or non-reactive state (Figure 1B). Mitochondria cannot synthesize GSH *de novo*, yet harbor their own GSH pools (∼10 mM) (Kojer et al., 2012; Ribas et al., 2014), imported from the cytosol (Mckernan et al., 1991). The presence of mitochondrial glutathione reductase, together with NADPH, permits reduction of GSSG to GSH and its recycling. Intriguingly, the IMS GSH pool depends on cytosolic import through porins in the OMM whereas the matrix GSH pool does not (Kojer et al., 2012), implying tight regulation by reduction enzymes (e.g., glutathione reductase), with little exchange outside of the matrix.

## Redox Control Mechanisms Through Transport at Mitochondrial Membranes

Though short-lived, ROS can exit the mitochondria to limit ROS accumulation or transmit signals to the extramitochondrial space. Spatially, ROS generated in the IMS are most likely to reach the cytosol where they function as signaling molecules or are degraded by catalases (predominantly in peroxisomes) to water and oxygen (Figure 1A). How can ROS pass through up to two lipid bilayer barriers whilst remaining intact and bypassing potential oxidative reactions? Since superoxide is charged and membrane diffusion of H_2_O_2_ is limited (Antunes and Cadenas, 2000; Cordeiro, 2014), membrane transporters are necessary and would offer protection from reactions. Anion channels could permit superoxide anions (Hawkins et al., 2007). In mitochondria, there are several anion channels (reviewed in Mackova et al., 2018). Though superoxide affects the activity of IMAC in the IMM and VDAC in the OMM, definitive evidence from electrophysiological studies for its conductance remains elusive.

Rather than direct superoxide conductance, superoxide could be dismutated to H_2_O_2_ by SOD2 and leave the matrix to the IMS by membrane diffusion or through AQP water channels (Figure 1A), so-called peroxiporins (Bienert et al., 2006; Bienert and Chaumont, 2014; Wang et al., 2020).

The major ROS species crossing the second membrane barrier between the IMS and cytosol is H_2_O_2_ (Figure 1A). The most obvious candidate for OMM H_2_O_2_ transfer is VDAC, since the OMM lacks AQPs, but no experimental evidence exists. A further consideration is the limited membrane diffusion of H_2_O_2_ that is rate limiting for release of ROS bursts to the cytosolic compartment, but possibly sufficient for signaling purposes. Why could H_2_O_2_ membrane diffusion occur at the OMM but not at the IMM? Cytosolic and matrix H_2_O_2_ concentrations have been estimated at 80 pM (Lim et al., 2015) and 5–20 nM (Sies and Jones, 2020), respectively, and H_2_O_2_ may concentrate at cristae junctions, similarly to protons, to enhance the diffusion concentration gradient between IMS and cytosol. Other factors to take into consideration for influencing increased OMM H_2_O_2_ permeability are differences in molecular makeup of the two mitochondrial membranes. For instance, lower protein to lipid ratio of the OMM could increase the diffusion surface area, OMM lipid composition could affect lipid fluidity, OMM lipid fatty acid chain length decrease diffusion distance, or looser packing of OMM lipids permits thoroughfare of small molecules (Comte et al., 1976; Bienert et al., 2006).

## Transport at OMM and IMM and Properties in Relation to Metal Ion Movement

The OMM and IMM are functionally linked to maintain intramitochondrial spaces of defined ionic and proteinaceous composition. Achieving this requires a high degree of control by various transporters that are mainly expressed in the IMM. The IMM is structurally and functionally separated into tubular invaginations called cristae and the inner boundary membrane; these juxtapose the OMM and form the peripheral space.

It is assumed that OMM permeability is solely governed by the existence of relatively large pores, partially represented by VDACs or porins, which constitute approximately 50% of OMM proteins, allowing unregulated passage of small solutes intended for the mitochondrial matrix. VDACs permit a variety of negatively and positively charged ions as well as small organic molecules and metabolites up to 5 kDa to pass the OMM and traverse the IMS to reach the matrix (Colombini, 2016). VDAC can switch between different open or half-open/closed conformations and alternate between anion and cation conductive states (Tan and Colombini, 2007). Although it is not quite clear how VDAC switches between these states, some regulatory mechanisms have been recently described (reviewed in Lee and Thévenod, 2020).

In contrast, the IMM is highly selective due to the presence of an array of transport proteins that tightly regulate access to the mitochondrial matrix and in the opposite way to the IMS. Ion movement across the IMM requires uniporters, symporters and antiporters. They include the mitochondrial calcium uniporter (MCU) complex (Kamer and Mootha, 2015; Mammucari et al., 2017), the mitoferrin (Mfrn) 1/2 uniporters carrying Fe (II), Cu (II), and Mn (II), SLC25A37 and SLC25A28 (Paradkar et al., 2009), the Cu (II) transporter SLC25A3 (Boulet et al., 2018) and K^+^ channels (Szabo et al., 2012; Augustynek et al., 2017). Furthermore, a K^+^/H^+^ exchanger (Zotova et al., 2010), various carriers for metabolites, including ANT (Palmieri and Monne, 2016), as well as translocases of the inner membrane (TIMs) that translocate proteins produced from nuclear DNA through these membranes for use by mitochondria (Pfanner et al., 2019) have been described.

The simplified view of OMM permeation involving VDAC has been challenged and complicated by recent reports identifying several OMM channel proteins and transporters (reviewed in Becker and Wagner, 2018), suggesting that the permeability of the OMM for electrolytes and small organic molecules is much more selective than previously thought. Moreover, we have identified the proton-coupled symporter DMT1 as a functioning OMM protein (Wolff et al., 2014a, 2018) (see section “A Recent, Versatile Candidate for OMM Import of Divalent Cations”).

Is tight control of ion entry at the OMM level necessary, considering the selective permeability of the IMM to ions? At least for redox active essential metal ions, such as Fe (II), Mn (II), and Cu (I), it would make sense to coordinate their uptake at the OMM with their rate of entry at the IMM to avoid accumulation of these potentially harmful Fenton metal ions in the IMS. One could argue, however, that metal ion-induced oxidative stress in the IMS space is negligible because ROS are constantly produced as byproducts of the ETC (Figure 1A). Nevertheless, excessive ROS formation in the IMS, such as by accumulation of redox active metal ions, increases cytC release from mitochondria. Notably, a regulated transport pathway for redox active metal ions in the OMM, such as DMT1, would minimize that hazard. Moreover, an electrogenic H^+^-driven transporter, such as DMT1 (Gunshin et al., 1997), at the OMM would represent an elegant mechanism to regulate uptake of Fe (II) for synthesis of Fe-sulfur clusters in the matrix that are required by the ETC complexes. Hence, the augmented proton-motive force generated by an increased ETC activity across the IMM would consequently slow down import of Fe (II) in a negative feedback loop.

## A Recent, Versatile Candidate for OMM Import of Divalent Cations

To end in heme, Fe must also eventually cross the mitochondrial IMM, transfer that occurs, at least in part, via Mfrns (Paradkar et al., 2009), but what about the gap in movement from endosomes to there? Fe could enter the detectable, but difficult to define, labile Fe pool next (Cabantchik, 2014); or it might directly enter the mitochondria. Descriptions of how Fe entered into mitochondria after DMT1 driven endosomal exit in the last decade ranged from “not well understood” (Chen and Paw, 2012) with speculation about roles for two Fe chaperones designated PCBP1 and PCBP2 (Philpott and Ryu, 2014) to apparently unconstrained entry through the OMM from an Fe donor (Lill et al., 2012). They also included an intriguing docking of the organelles called “kiss and run” (Sheftel et al., 2007; Richardson et al., 2010; Hamdi et al., 2016), reviewed recently (Lane et al., 2015; Paul et al., 2017). Possibilities for the large pore that would be needed for entry include the (actually selective) VDAC or other porins (Colombini, 2016). The issue for iron can also be placed in the larger context of how selectively the OMM and IMM perform as barriers – debated above in section “Transport at OMM and IMM and Properties in Relation to Metal Ion Movement.” We considered another mechanism when we detected mitochondrial DMT1 (Wolff et al., 2014b) closely associated with OMM markers and confirmed by subcellular fractionation. Additional support quickly followed (Wolff et al., 2014a), with both works reviewed subsequently (Thévenod and Wolff, 2016). Later evidence showed that DMT1 was involved in import of Fe^2+^ and Mn^2+^ across the OMM (Wolff et al., 2018).

The initial paper (Wolff et al., 2014b) relied on a yeast-2-hybrid system to show that mitochondrial proteins, Tom6 and COXII, were potential DMT1 interaction partners. Co-immunoprecipitation of the latter with DMT1, and knowing that Parkin also did so (Roth et al., 2010), implicated DMT1 as a mitochondrial protein. DMT1 immuno-reactivity was detected in rat kidney cortex mitochondria by immunogold labeling (Wolff et al., 2014a). Mitochondria isolated from cultured epithelial cells and stably DMT1-transfected cells exhibited DMT1 immunoreactivity in Western blots that was substantially reduced or undetectable after stripping of the OMM; while DMT1 was enriched in OMM over mitochondria in images (Wolff et al., 2014b).

Experimental data in the latest paper (Wolff et al., 2018) indicated that increasing OMM DMT1 led to increased Fe uptake in isolated mitochondria by several criteria. Hence, DMT1 can alleviate rate-limited entry, ruling out unimpeded diffusion into mitochondria. Importantly, mitochondria isolated from kidneys of homozygous (*b/b*) Belgrade rats with a G185R mutation in DMT1 that reduces its transport capabilities had severely diminished uptake of Mn^2+^ in comparison to mitochondria from +/+ rats (with ones from +/*b*, intermediate), indicating that mitochondrial Mn^2+^ import is mostly dependent on DMT1. Two inhibitors of DMT1 blocked its activity in isolated mitochondria. While import of Mn^2+^ exhibited strong DMT1 dependence by several criteria, Fe^2+^ entry appeared to depend on not only DMT1 but also another route that currently remains unidentified.

Divalent metal transporter 1 is an H^+^-cotransporter (Gunshin et al., 1997), a property that raises the role of pH gradients in mitochondria. Import of Mn^2+^ exhibited pH dependence (Wolff et al., 2018) like that found before (Garrick et al., 2006a). DMT1-dependent Fe^2+^-induced quenching of an Fe indicator dye (Wolff et al., 2018) was stimulated by an inward-directed proton gradient (pH 6.2_*o*_ vs. pH 7.0_*i*_) relative to an opposite pH gradient (pH 7.6_*o*_ vs. pH 7.0_*i*_) with quenching unaffected for mitochondria equilibrated at pH 6.2 or 7.6, excluding an unspecific pH effect. Interestingly, no such pH gradient dependence has been observed for the IMM Fe carrier Mfrn1 from the cichlid *Oreochromis niloticus* reconstituted in proteoliposomes (Christenson et al., 2018). Moreover, Mfrn1-mediated Fe uptake was substantially diminished below an external pH of 6.5. Although this value is lower than what has been determined for the IMS (pH 6.88) in mitochondria from an endothelial cell line (Porcelli et al., 2005), IMS pH in cells *in vivo* may well be lower due to stronger dependence on mitochondrial energy metabolism (cf. below). Thus, DMT1 and Mfrns may conceivably cooperate in Fe^2+^ delivery to the mitochondrial matrix relying on acidic IMS pH.

Although some might consider the descriptions for OMM entry to be mechanistic alternatives, we note that one (Chen and Paw, 2012) was really a statement acknowledging the field’s status; while another (Lill et al., 2012) can be treated as implying that absent a candidate for a gateway is equivalent to the absence of a gateway. We regard it as nearly self-evident that an organelle that manages O_2_, unavoidably generates ROS and needs transition metals to carry out its functions necessarily regulates entry of their ions. The third description (Richardson et al., 2010) clearly includes regulation. Transient docking of endosomes and mitochondria that allows Fe to move from the Tf-Tf receptor complex to within the intermembrane space of the mitochondria without a presence in the cytosol, can also involve DMT1 on the OMM. One could jokingly in part refer to DMT1, also present on the endosomal membrane, as potentially the lips of the kiss by considering the possibility that DMT1 on the surface of the two organelles associates to help docking occur. This mechanism would provide a tunnel so that Fe^2+^ does not exist in a state where it is freely exposed to the cytosol in transit. It is important to note that participation of DMT1 in import of metal ions into mitochondria and argument for docking of the two organelles rely on independent observations and either mechanism could be validated or challenged without the other hypothesis necessarily rising or falling as well. For example, “kiss and run” receives strong support in erythroid precursors where heme biosynthesis demands high Fe flux (Hamdi et al., 2016) but the strongest non-erythroid evidence supporting the hypothesis comes from experiments on MDCK-PTR cells (Das et al., 2016). Blocking intra-endosomal Fe release increased both duration of the two organelles’ interactions and motility of Tf-endosomes, which may provide inspiration for experiments on whether OMM DMT1 participates in the same process. Although this cell line was derived from a canine kidney, its use is primarily noteworthy for showing that erythroid commitment to extraordinary Fe flux into heme need not be a requirement for organelle docking to prevent ROS. One virtue of coordinating the two hypotheses to make DMT1 the lips of the kiss is that the endosome will expel protons to drive Fe^2+^ or Mn^2+^ across its membrane into the OMM and perhaps through Mfrn into the mitochondrial matrix.

## How Does DMT1 Integrate Into Mitochondrial Metal Homeostasis?

The data referenced (Wolff et al., 2014a, 2018) support a role for DMT1 in import of Fe^2+^ and Mn^2+^ across the OMM; however, much more needs to be learned to integrate it fully into how the organelle handles metal ions. While DMT1 probably accounts for most Mn influx, an unidentified participant adds to Fe influx. Because DMT1 can handle multiple other metals (Gunshin et al., 1997; Garrick et al., 2006b; Illing et al., 2012), identifying for which ones its role is significant at the OMM remains to be determined. Hence, integration for many metals can only be speculative. Nevertheless, we must add Cd, a toxic metal with a high DMT1 affinity as relevant for this review. Presumably, Cd toxicity derives from its not having Fenton chemistry, with it usually considered not to be a transition metal.

A substantial portion of metal metabolism occurs within the IMM after DMT1 transport across the OMM. Mfrn 1/2 clearly take over for Fe^2+^ (Chen and Paw, 2012) at the IMM with the former involved in Fe crossing erythroid IMMs while its paralog does this for non-erythroid IMMs. Mfrn 1 is capable of transporting Mn and some other DMT1 substrates (Christenson et al., 2018) but whether it or its counterpart play that role at the IMM is not yet known. Another question that having DMT1 control movement of divalent metals across the OMM raises relates to the metals’ role in generating and managing free radicals. DMT1 binds the Fe chaperone PCBP2 (Yanatori et al., 2014), transferring Fe directly to it. Is the IMS insubstantial in metal homeostasis? Or is there a need for chaperones like PCBP1 or 2? For Cu the presence of SOD1 and multiple other Cu-related proteins there (Baker et al., 2017) sets a precedent that may well apply to other members of the metallome.

**FIGURE 2:**
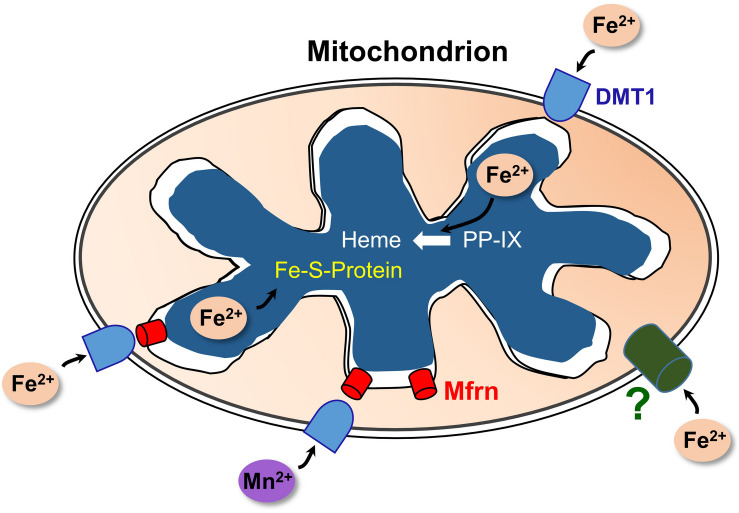
A model of DMT1 participating in mitochondrial iron homeostasis. Keys: DMT1, mitoferrins (Mfrn), ferrous iron (Fe^2+^), Mn^2+^, iron-sulfur cluster proteins (Fe-S-Protein), protoporphyrin IX (PPIX), and heme. Fe^2+^ enters the OMM via DMT1 and an as-yet unidentified pathway (?); while Mn^2+^ apparently relies primarily on DMT1 for import. After entry, the two cations could enter the IMS then pass through Mfrn (lower right) or move directly through Mfrn (lower left). Adapted and redrawn with permission from Garrick and Thévenod (2019).

Selected aspects of how DMT1 could participate in mitochondrial metal metabolism are represented in Figure 2. Other aspects, omitted to avoid making the representation too complicated, include the influence of proton gradients, possible coordination with endosomal docking to mitochondria that might create a tunnel from endosomes to mitochondrial matrix, possible chaperones in the IMS and potential distinctions between occurrences in renal mitochondria and other specialized mitochondria like erythroid ones.

## Systemic and Cellular Iron Homeostasis

**FIGURE 3:**
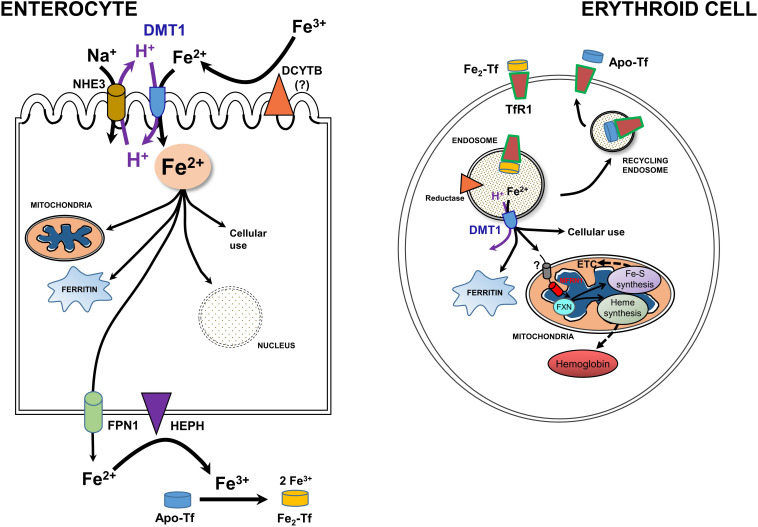
A model for integration of cellular iron homeostasis with systemic iron homeostasis. Keys: Enterocytes, iron (Fe^3+^ & Fe^2+^), a ferrireductase (DCYTB); “?” indicates that it is unlikely to be the only reductase), DMT1, NHE3, a mitochondrion, ferritin, the nucleus, Hephaestin (HEPH), Ferroportin (FPN1) and Na^+^/K^+^ antiporter. Not shown are tight junctions with other enterocytes and the Tf-receptor that likely binds apo-Tf so that it can be loaded with Fe. Also keyed: Erythroid cells, TfR1. di-ferric Tf binding to TfR1, apo-Tf released by it, endocytosis -making an endosome to go through the Tf-cycle (see text), an endosomal reductase, an ATPase, DMT1, cytosolic ferritin, mitochondrial components, and metabolism including Mfrn1 = erythroid mitoferrin, FXN – frataxin, ETC and Fe-S plus Heme synthesis and hemoglobin formation. Adapted and redrawn from Wang et al. (2019) with permission from Oxford University Press on behalf of the American Society for Nutrition.

Newly recognized mitochondrial (including renal mitochondria) acquisition of iron via OMM DMT1 needs to be set the broader context of systemic and cellular iron homeostasis (Hentze et al., 2010; Garrick, 2016; Wang et al., 2019; Yanatori and Kishi, 2019). Systemic iron metabolism in humans begins with uptake of iron from the diet in the duodenal lumen where it presents as Fe^3+^ (Figure 3, left). Hence, a ferrireductase must generate Fe^2+^ with DCYTB, representing Duodenal Cytochrome B, also known as cytochrome b reductase 1, encoded by CYBRD1. DMT1 is the major Fe^2+^ importer, acting as a proton symporter. The inward proton gradient is maintained by NHE3, a Na^+^/H^+^ antiporter. Arriving in the enterocyte, a polarized epithelial cell, Fe^2+^ follows two main pathways: either vectorial for systemic distribution, i.e., transcellularly to exit (the main iron-related function as the source of nearly all systemic Fe), or cellular by entering the mitochondrion or nucleus, storage in ferritin, or other cell usages. Chaperones like PCBP’s aid in minimizing ROS. Fe^2+^ exit depends on oxidation to Fe^3+^ by Hephaestin (HEPH) or possibly other ferroxidases coordinated with export by Ferroportin (FPN1); Fe^3+^ then interacts with apo-Tf to load the Tf to mono- or di-ferric Tf in the circulation, representing Tf bound Fe (TBI).

Cellular iron homeostasis is well embodied by erythroid precursors because a substantial majority of iron acquisition leads to hemoglobin synthesis (Figure 3, right). They incorporate Fe delivered by Tf from the circulation. Fe-Tf or Fe_2_-Tf bind to the ubiquitous Tf-receptor 1 (TfR1) that is present on the surface of immature erythroid and other cells where the complex undergoes endocytosis. The endosome formed by this process acidifies due to the activity of a vacuolar H^+^-ATPase, supplying protons to allow the Tf-TfR1 complex to release Fe^2+^ in cooperation with endosomal reductase. Relieved of its Fe, apo-Tf-TfR1 exocytosis to the cell surface; at the higher pH there, the complex releases apo-Tf to the circulation to retrieve more Fe. The H^+^ also drive DMT1 as an endosomal exit transporter to supply the iron to mitochondria as discussed in sections “A Recent, Versatile Candidate for OMM Import of Divalent Cations” and “How Does DMT1 Integrate Into Mitochondrial Metal Homeostasis?” The vast majority of Fe flux in erythroid mitochondria goes into heme synthesis needed for hemoglobin formation, but non-erythroid cells have a more even balance between heme synthesis and non-heme iron utilization such as in iron-sulfur proteins. For its cellular regulation, iron depends largely on an iron response element-iron regulatory protein system (IRE-IRP) (reviewed in Hentze et al., 2010; Papanikolaou and Pantopoulos, 2017).

When mature erythrocytes become effete, nearly all their iron is recovered by macrophage (or hepatic Kupffer cells) that may store it temporarily, but ultimately resupply most of it to erythroid precursors. The liver also serves as a temporary depot via the portal circulation and TBI constitutes the usual physiological source of iron for most cells where metabolism resembles the erythroid cell except that demand for heme biosynthesis is modest. With no regulated means for iron depletion, excellent renal recovery (see below) and competent recycling, the onus is on the duodenum (and recovery systems) to prevent systemic iron overload and to withhold iron during inflammation. How hepcidin, a master regulatory peptide, accomplishes these goals is well covered in reviews (Hentze et al., 2010; Wang et al., 2019; Camaschella et al., 2020).

**FIGURE 4:**
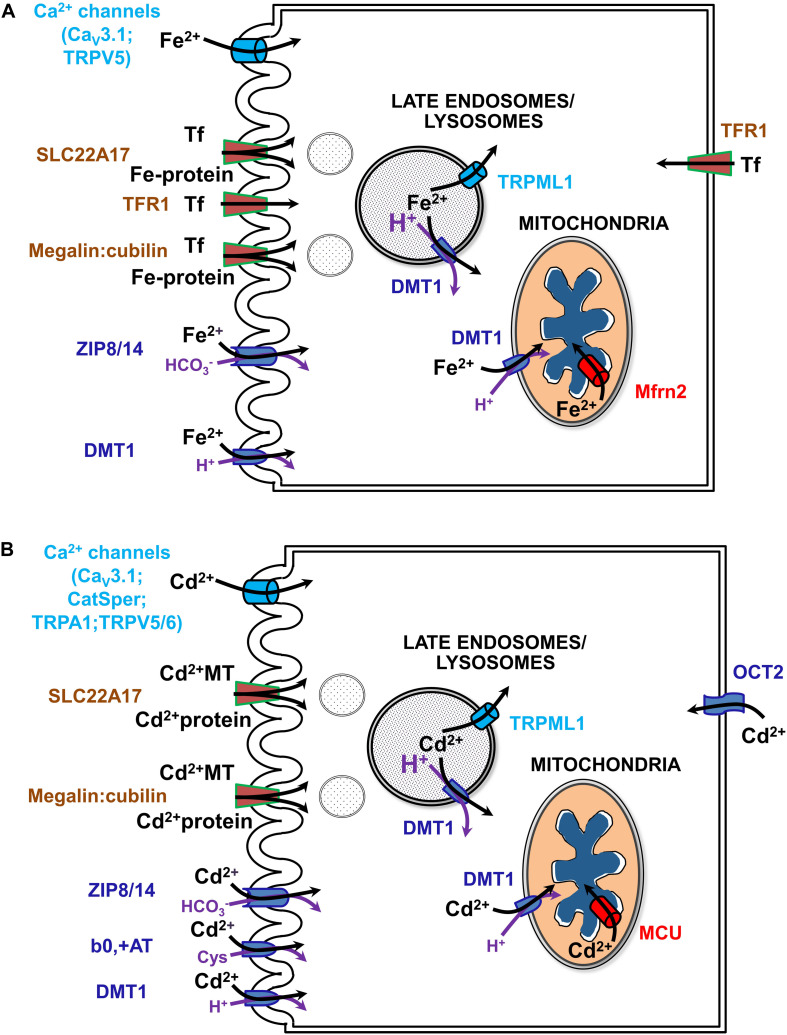
Uptake pathways for Fe **(A)** and Cd **(B)** via channels, solute carriers (SLCs) and receptors in renal epithelial cells. See text and (Thévenod and Wolff, 2016; Thévenod et al., 2019; Van Swelm et al., 2020) for further details. b0,+AT, amino acid/cystine transporter (SLC7A9/SLC3A1); CatSper, cation channel of sperm; Cav3.1, voltage-gated calcium channel alpha subunit 3.1; DMT1, divalent metal transporter 1 (SLC11A2); MCU, mitochondrial calcium uniporter; megalin:cubilin, the megalin:cubilin:amnionless complex; Mfrn2, mitoferrin 2 (SLC25A28); OCT2, organic cation transporter 2 (SLC22A2); SLC22A17, NGAL/lipocalin 2 receptor; Fe-Tf, holo-transferrin; TfR1, Tf receptor 1; TRPA1, transient receptor potential cation channel subfamily A member 1; TRPML1, transient receptor potential cation channel subfamily ML member 1; TRPV5/6, transient receptor potential cation channel subfamily V members 5/6; ZIP8/14, zinc transporters 8/14 precursors (SLC39A8/A14).

Much of the current review concerns renal management of systemic iron metabolism by recovering nearly all filtered forms of iron. Indeed, the kidney has emerged as an important physiological organ that contributes to regulation of systemic Fe levels (Smith and Thévenod, 2009). In addition to usage of reabsorbed Fe in cellular homeostasis, polarized epithelial kidney cells prevent Fe loss from the body by vectorial transport of loosely bound Fe and by receptor-mediated endocytosis of high- and low-affinity Fe-binding proteins (such as Tf or albumin, respectively) into the blood (Langelueddecke et al., 2012; Smith et al., 2019) (reviewed in Thévenod and Wolff, 2016). Fe transport mechanisms have also been identified in the kidney that depend on systemic and/or locally produced hepcidin levels as well as on IRE-IRP system dependent and independent transporters (reviewed in Thévenod and Wolff, 2016; Van Swelm et al., 2020; see Figure 4A).

## Fe Overload and Renal Injury

Disturbances in cellular and systemic Fe balance contribute to kidney injury either by initiating oxidative stress and mitochondrial dysfunction or by modulating inflammatory processes (Thévenod and Wolff, 2016; Van Swelm et al., 2020). Fe overload of the kidney occurs by increased filtration of Fe, in the form of TBI and NTBI, either due to systemic Fe overload or kidney disorders. Hemochromatoses and dyserythropoiesis underlie mostly systemic Fe overload of genetic origin whereas acquired systemic Fe overload usually occurs through transfusions (Brissot et al., 2019). Renal diseases causing Fe overload of the kidney involve proteinuria of glomerular origin, that predominantly causes PT damage, or decreased PT reabsorption (e.g., as in Fanconi syndrome due to PT damage) with Fe overload of downstream nephron segments. Overload occurs because the PT (in particular the S1-segment) (Schuh et al., 2018) is equipped with both a receptor for (metallo-)protein endocytosis, megalin:cubilin:amnionless (Kozyraki et al., 2001; Weyer et al., 2011) and endo-lysosomal machinery expressing DMT1 (Abouhamed et al., 2006), promoting Fe accumulation and damage of PT cells. Distal nephron segments take up Fe via DMT1 in their apical membranes (DCT > thick ascending limb of LOH > CD) (Ferguson et al., 2001). The protein receptor SLC22A17 [with higher affinity to protein ligands, such as Tf, neutrophil gelatinase-associated lipocalin (NGAL) / lipocalin-2 and albumin, than megalin:cubilin:amnionless] engulfs Fe-protein complexes in DCT and CD (Langelueddecke et al., 2012). Increased uptake of Fe via these transporters and receptors may induce nephrotoxicity (Thévenod and Wolff, 2016; Van Swelm et al., 2020). Fe overload has been described in the distal nephron of hemochromatosis mouse models (Moulouel et al., 2013). Hemolysis or rhabdomyolysis can also lead to increased glomerular filtration of heme, hemoglobin or myoglobin, which are taken up by both PT and the distal nephron (Moulouel et al., 2013) via megalin:cubilin:amnionless (Gburek et al., 2002) and SLC22A17 (Van Swelm et al., 2018). Interestingly, urinary tract infection decreases Fe accumulation in the distal nephron of hemochromatosis mouse models (Houamel et al., 2016) along with decreased SLC22A17 expression (Betten et al., 2018), supporting a role for SLC22A17 in mediating Fe uptake in the distal nephron. In addition to apical exposure, tubular cells should also be exposed to increased circulating Fe levels at their basolateral side during Fe overload conditions.

Importantly, alterations of cellular Fe homeostasis pathways involving accumulation of damaging redox active labile Fe in relevant nephron segments may elicit Fe-mediated renal cell injury or death and play a causative role in the pathogenesis of AKI (and possibly CKD) (reviewed in Scindia et al., 2015; Swaminathan, 2018; Van Swelm et al., 2020). Necroptosis (Van Swelm et al., 2018) and possibly two other forms of regulated cell death, ferroptosis and pyroptosis, have been implicated in AKI induced by Fe (reviewed in Van Swelm et al., 2020), but evidence is weak here, and no data are available for the mode(s) of cell death involved in CKD. Fe-dependent AKI may result from increased renal exposure to heme, myoglobin and/or from ischemia–reperfusion injury because release of catalytic Fe from damaged renal tubular epithelial cells into the tubule lumen may result in exposure of neighboring cells. Fe either initiates oxidative stress with ROS formation and mitochondrial dysfunction, mainly by inducing the mPTP (see sections “Apoptosis Signaling” and “Cd and Kidney Mitochondrial Damage”) or is a potent modulator of inflammation. Persistence of these harmful pathological processes is thought to drive AKI- to-CKD progression (Liu et al., 2018), which is supported by the observation of increased accumulation of Fe in tubular epithelial cells and infiltrating macrophages in animal models of CKD and in kidney biopsy samples from patients with CKD (reviewed in Van Swelm et al., 2020). Moreover, clinical studies indicate an association between Fe overload and CKD (Slotki and Cabantchik, 2015) that may involve mitochondrial damage (see section “Fe and Mitochondrial Damage”).

## Fe and Mitochondrial Damage

Kidney tubular epithelial cells are vulnerable to oxidative stress due to their energy demand and high numbers of mitochondria (Liu et al., 2018). Yet data on damaging effects of Fe on renal mitochondria are scarce (Figure 1B). Several models of AKI by acute Fe overload of the kidney show consistently damaged kidney mitochondria induced by increased mitochondrial Fe and oxidative stress. Twenty hours after a single intraperitoneal injection of iron-dextran (500 mg/kg body weight) into rats, increased mitochondrial Fe correlated with increased lipid peroxidation and decreased mitochondrial α-tocopherol content in cortex and medulla (Galleano et al., 1994), whereas other mitochondrial functions were unaffected. Thirty minutes to 3 h after intraperitoneal injection of Fe- nitrilotriacetic acid (10 or 20 mg Fe/kg body weight), mitochondria of rat kidney PT cells showed increased oxidative damage, supported by accumulation of 4-hydroxy-2-nonenal-modified proteins, indicating oxidative breakdown of polyunsaturated fatty acids and related esters (Zainal et al., 1999). In rat kidneys, myoglobin Fe released into the circulation by experimental rhabdomyolysis produces oxidative stress and mitochondrial dysfunction through lipid peroxidation of the mitochondrial membranes (Plotnikov et al., 2009). Inhibitors of mPTP, mitochondria-targeted antioxidant (SkQ1), and Fe chelation with deferoxamine abrogated release of cytC as well as defective ETC and OXPHOS. In a subsequent study of myoglobinuric AKI in rats, cell death occurred with sustained caspase 3 cleavage (Funk and Schnellmann, 2012). Expression of mitochondrial fission protein Drp1 and the fusion protein Mfrn2 were elevated as well as markers of mitochondrial biogenesis (PGC-1α and PGC-1-related co-activator). Respiratory proteins NADH:Ubiquinone Oxidoreductase Subunit B8, ATP synthase β, cytC oxidase subunit I (COX I), and COX IV were decreased, indicating persistent disruption of mitochondrial homeostasis even in the presence of mitochondrial recovery signals (Funk and Schnellmann, 2012). Postoperative AKI caused by hemoglobinemia, has also been associated with an enhanced oxidative stress response and lipid peroxidation (Billings et al., 2011).

Fe-induced excessive formation of mtROS and lipid peroxidation inhibit ETC activity (Musatov and Robinson, 2012). In addition, increased ROS formation depletes mitochondrial antioxidants, such as GSH, or damage anti-oxidative defense enzymes, as described in liver mitochondria (Jagetia and Reddy, 2011; Figure 1B). Clinical studies indicate an association between Fe overload and CKD (Slotki and Cabantchik, 2015). Mitochondrial damage induced by increased Fe has been implicated in the pathophysiology of CKD. Damage involves a decrease in frataxin, a mitochondrial protein possibly involved in assembly of Fe-sulfur clusters and acting as an Fe-binding protein (Pastore and Puccio, 2013). Moreover, accumulation in mitochondria of 5-aminolevulinic acid, a heme precursor that increases oxidative stress and has direct disruptive effects on mitochondrial function has been discussed (reviewed in Nakanishi et al., 2019). Nevertheless, the evidence linking these molecular pathologies with CKD is correlative.

Interestingly, loss of SOD2 activity culminates in Fe accumulation in mitochondria accompanied by increases in regulators of the Fe pool: ferritin, transferrin receptors, transferrin, hepcidin, and frataxin (reviewed in Holley et al., 2011). Furthermore, SOD2 knockdown in erythroid cells results in reduction of the IMM transporter ABCB7 (Martin et al., 2011), which exports Fe-sulfur centers to the cytosol for synthesis of Fe-sulfur center containing proteins. Moreover, ABCB7 downregulation results in mitochondrial Fe overload as well as diminished SOD2 activity (Cavadini et al., 2007). The main alterations of mitochondrial structure, function and expression elicited by increased mitochondrial Fe are summarized in Table 1 (left).

**TABLE 1:** Key alterations of mitochondrial structure, function and expression elicited by increased mitochondrial Fe and Cd transport.

	**Fe**		**Cd**	
Oxidative damage (lipids; proteins)	**+**	Galleano et al., 1994; Zainal et al., 1999; Plotnikov et al., 2009; Billings et al., 2011; Musatov and Robinson, 2012	**+**	Reviewed in Lee and Thévenod, 2020
Depletion of antioxidants (GSH; α-tocopherol)	**+**	Galleano et al., 1994	**+**	Reviewed in Lee and Thévenod, 2020
Damage of antioxidative enzyme defense (↓ function and/or expression)	n.d.		**+**	Reviewed in Cuypers et al., 2010
Disruption of ETC, OXPHOS and Δψ_*m*_	**+**	Plotnikov et al., 2009; Musatov and Robinson, 2012	**+**	Belyaeva et al., 2004; Wang et al., 2004; Lee et al., 2005a,b
Mitochondrial permeability transition pore (mPTP)	**+**	Plotnikov et al., 2009	**+**	Belyaeva et al., 2002, 2004
Activation of apoptotic signaling	**+**	Plotnikov et al., 2009; Funk and Schnellmann, 2012	**+**	Lee et al., 2005a,b; Nair et al., 2015
Changes in fusion-fission dynamics and biogenesis	**+**	Funk and Schnellmann, 2012	**+**	Nair et al., 2015
Alterations of mitochondrial DNA levels	n.d.		**+**	Nair et al., 2015
Decreased expression of respiratory proteins	**+**	Funk and Schnellmann, 2012	**+**	Reviewed in Lee and Thévenod, 2020
Decreased heme and Fe-sulfur cluster synthesis	**+**	Pastore and Puccio, 2013; reviewed in Nakanishi et al., 2019	n.d.	
Structural and membrane defects	**+**	Zainal et al., 1999	**+**	Kerek and Prenner, 2016; Reviewed in Lee and Thévenod, 2020

*Observed effects are also dependent on time of exposure and metal ion concentrations used. For additional details and references, see sections “Fe and Mitochondrial Damage” and “Cd and Kidney Mitochondrial Damage.” n.d., not determined.*

## Cd Toxicity and the Kidney

Due to enhanced industrial and agricultural activities, Cd accumulates in the environment. Hence, Cd is one of the top 20 hazardous substances worldwide and a significant public health issue (Faroon et al., 2012). Further, chronic low Cd exposure (CLCE) is a health hazard for ∼10% of the world population with increased morbidity and mortality (Jarup and Akesson, 2009; Moulis and Thévenod, 2010).

Food and cigarette smoking are the prime sources of CLCE (Satarug and Moore, 2004). For non-smokers, food grown in Cd-containing rock phosphate fertilizers is the major source of CLCE (Pan et al., 2010). Bioaccumulation of Cd in plants, including tobacco, is the first step in the human food chain that results in Cd accumulation in the body. Various organs and systems are affected by CLCE, causing kidney damage, osteoporosis, genotoxicity, teratogenicity, or endocrine, and reproductive defects (Jarup and Akesson, 2009). Cd in tobacco smoke is an independent risk factor in the development of smoking-associated chronic diseases, such as cardiovascular disorders and cancer (Abu-Hayyeh et al., 2001; Huff et al., 2007).

The kidneys contain ∼60% of the Cd body burden in the age range of 30–60 years (Salmela et al., 1983; Jarup and Akesson, 2009). Cd accumulates in the kidneys, in particular the PT, because intracellular Cd induces the upregulation of the protein MT, rich in sulfhydryl (SH) groups that bind the metal ion with high affinity and thereby prevent its toxic effects (Freisinger and Vasak, 2013). Although Cd buffering by MT is initially protective for the kidneys, it in fact represents a dangling sword of Damocles because Cd accumulation over time develops into an endogenous source of high concentrations of potentially toxic Cd and is associated with chronic kidney disease (Gibb et al., 2019; Satarug et al., 2020).

The question remains how Cd enters mammalian cells in general, and particularly kidney cells, because no physiological process requires the non-essential toxic-only ion Cd. Simply, Cd imitates essential metal ions such as Fe, Mn, Zn, Ca, and Cu, and crosses cell membrane barriers by competing for their transport pathways (Thévenod et al., 2019; see also Figure 4B). To describe this process, the term “ionic and molecular mimicry” has been created (Clarkson, 1993; Bridges and Zalups, 2005). Nevertheless and unfortunately, Cd ionic mimicry does not hold for the only known cellular Fe efflux transporter FPN1 (Mitchell et al., 2014) which is expressed basolaterally in kidney PT cells (Wolff et al., 2011) and does not allow Cd to exit renal cells. The inability of FPN1 to transport Cd for vectorial reabsorption into the circulation (in contrast to Fe, Co, and Zn) may partly explain major Cd accumulation and toxicity of renal cortical PT cells (see above).

By binding to essential side groups of biomolecules (e.g., SH groups) and/or displacing essential metals from macromolecules, Cd disrupts cellular functions with subsequent death or disease (Moulis, 2010; Thévenod and Lee, 2013). Cd interacts with proteins by substituting for zinc ions, as in enzymes or transcription factors (Maret and Moulis, 2013; Petering, 2017), or replacing calcium in cellular signal transduction, interfering with thiol-dependent redox systems, and modifying second messenger levels, growth and transcription factors (Thévenod, 2009; Thévenod and Lee, 2013). Cd is not a transition-metal ion undergoing Fenton chemistry, yet in biological systems, it indirectly increases ROS and RNS. Cd depletes endogenous anti-oxidative ROS scavengers (such as GSH), affects ROS-producing/metabolizing anti-oxidative enzymes, disrupts mitochondrial function, in particular the mitochondrial ETC (see also Figure 1C), and/or displaces redox active metals, such as Fe or copper (Cuypers et al., 2010). Cd damages DNA indirectly by causing increased ROS formation and therefore has been classified as a class I carcinogen; it also interferes with major DNA repair systems and inactivates tumor suppressor functions by targeting zinc-finger proteins. This effect may cause genomic instability and promote tumor initiation and progression (Hartwig, 2013).

## Cd and Kidney Mitochondrial Damage

Mitochondria not only traffic and redistribute within the cell in response to local energy demands, but are also dynamic organelles that continuously fuse and divide to maintain functional mitochondria when cells experience metabolic or environmental changes (Youle and Van Der Bliek, 2012). Moreover, chronic stress and high metabolic demand induce mitochondrial biogenesis, which is partially regulated by the transcription factors peroxisome proliferator-activated receptor (Ppar) and its coactivator 1 (Pgc-1) (Scarpulla, 2012; Dorn, 2019).

Cd damages mitochondrial structures and membranes in kidney cortex by reducing cristae number, inducing cristae shortening and reduced expression of COXs (reviewed in Lee and Thévenod, 2020). Cd interacts with cardiolipin, a mitochondrial phospholipid localized in the IMM, in artificial liposomes, increasing membrane rigidity (Kerek and Prenner, 2016), and thereby possibly enhances cytC release for apoptotic signaling.

Cd affects renal mitochondrial biogenesis concentration dependently, both *in vitro* and *in vivo* (Nair et al., 2015). At 1-10 μM CdCl_2_ for 24 h, Pparγ and mitochondrial DNA (mtDNA) were augmented in cultured renal PCT cells whereas Pparα and Pgc-1β were not affected, occurrences correlated with minor or low loss of GSH and low rates of apoptosis. In contrast, 30 μM CdCl_2_ attenuated Pparα, Pgc-1β and mtDNA, despite sustained Pparγ increase, and was associated with increased oxidized GSH GSSG and pro-apoptotic markers. Similarly in sub-chronically CdCl_2_-treated rats (1 mg/kg/day, s. c., 2 weeks), Pparα and mtDNA significantly increased whereas GSH was unchanged compared to saline-treated controls (Nair et al., 2015), indicating that mitochondrial biogenesis is part of an adaptive mechanism to chronic oxidative stress by Cd.

How does Cd permeate mitochondria? Cd may mimic calcium at binding sites due to their similar hydrated ionic radii (Marcus, 1988). Hence Cd could permeate the OMM via VDAC in its half-open/closed state, similarly as described for calcium (Colombini, 2016). Alternatively, Cd may cross the OMM through DMT1, given that it is a known substrate (Thévenod et al., 2019) (see sections “A Recent, Versatile Candidate for OMM Import of Divalent Cations” and “How Does DMT1 Integrate Into Mitochondrial Metal Homeostasis?”). Because Cd permeates certain types of Ca^2+^ channels, e.g., voltage gated T-type calcium channels or TRPV5/6 (reviewed in Choong et al., 2014), Cd permeation of the IMM may occur through the MCU complex, the major IMM Ca^2+^ channel (Kirichok et al., 2004; Thévenod et al., 2019). Indeed, as shown in isolated mitochondria from kidney cortex (as well as from liver) pharmacological MCU inhibitors ruthenium red, Ru360 or La^3+^ abolished Cd-induced mitochondrial dysfunction, such as swelling, loss of membrane potential and pro-apoptotic cytC release, consistent with Cd transport through the MCU into the matrix (Dorta et al., 2003; Li et al., 2003; Lee et al., 2005a,b).

To adjust to cellular energetic demands, mitochondria swell, and contract through monovalent cation cycling to regulate matrix biochemical reactions, such as β-oxidation, Krebs cycle activity and respiration that are decreased by mitochondrial swelling (Lizana et al., 2008; Nowikovsky et al., 2009). Isolated energized rat kidney cortex mitochondria suspended in KCl buffer undergo swelling followed by rapid contraction after addition of 5–20 μM CdCl_2_, whereas non-energized mitochondria do not contract (Lee et al., 2005b). Using pharmacological inhibitors, we showed that Cd entered via the MCU and elicited Δψ_*m*_ driven K^+^ influx via a K^+^ uniporter to induce matrix swelling, with subsequent activation of a quinine-sensitive K^+^/H^+^-exchanger, resulting in mitochondrial contraction (prior to dissipation of Δψ_*m*_) (Lee et al., 2005b). Hence, transient mitochondrial swelling by Cd may be part of an adaptive stress response, together with a temporary switch in energy metabolism, that precedes mitochondrial dynamics and removal of damaged mitochondria by mitophagy.

Once in mitochondria, Cd interferes with the function of the ETC to dissipate Δψm (Belyaeva et al., 2004; Lee et al., 2005b; Figure 1C). Cd (20 μM) inhibited ETC complex activities from various organs (CIII > CII >CI > CIV), maximally by ∼75% (Wang et al., 2004). CIII catalyzes the transfer of electrons from ubiquinol to cytC, and Cd competes at the zinc-binding site, preventing electron transfer and resulting in increased superoxide.

Intriguingly, CI can produce ROS through reverse electron transfer (RET) during low ATP demand and a large proton-motive force can drive electrons to flow to CI, leading to superoxide formation. RET depends on the proton-motive force and oxidative status of mitochondrial coenzyme Q and NADH pools (Robb et al., 2018). Could RET be relevant to Cd-induced oxidative stress? ETC inhibition by Cd will hinder electron shuttling, increasing NADH oxidation (Cameron et al., 1986) as well as dissipating Δψ_*m*_. These would result in either loss of the reduced NADH pool and/or matrix-directed proton-motive force, and quite plausibly drive RET and superoxide production through CI. These effects could enhance the loosely bound cytC pool and result in increased cytC apoptogenicity (Petrosillo et al., 2003). Loss of ATP generation by Cd through the ETC could be an important additional step in the transformation progression of normal cells, ultimately making the switch to glycolysis, also known as the Warburg effect, which is a hallmark of cancer cells (Gogvadze et al., 2009).

Similar to other apoptotic stimuli Cd dissipates Δψ_*m*_, possibly involving the mPTP, resulting in mitochondrial dysfunction and apoptosis (see section “Apoptosis Signaling”). Uncertainty about the molecular signature of mPTP is paralleled by conflicting studies addressing the question of whether Cd induces mPTP opening as part of its cell death signaling (Belyaeva et al., 2002; Li et al., 2003; Lee et al., 2005a). No evidence for Cd induction of mPTP was found in studies on Cd-induced swelling of isolated mitochondria from rodent kidney monitored by light scattering measurements in combination with known pharmacological modulators of the originally postulated mPTP components (cyclosporin A, bongkrekic acid, atractyloside) (Li et al., 2003; Lee et al., 2005a). Ineffectiveness of pharmacological modulators, such as cyclosporin A, in preventing Cd-induced swelling of isolated kidney and liver mitochondria, indicates that the mPTP is not “the” ubiquitous mitochondrial swelling mechanism elicited by Cd (reviewed in Lee and Thévenod, 2006). Indeed, opening of an IMM water channel, AQP8, by Cd is a likely entryway of water influx into the matrix to cause swelling of kidney (and liver) mitochondria (Calamita et al., 2005; Lee et al., 2005a; Lee and Thévenod, 2006). Interestingly, mammalian AQP8 appears to have the largest H_2_O_2_ permeability (Bienert et al., 2007). Genetic manipulation of mitochondrial AQP8 supports its role in H_2_O_2_ release to the cytosol (Danielli et al., 2019a,b), which could contribute to Cd-induced cell death.

Cd could activate mitochondrial AQP8 directly by binding at calcium binding sites found on AQPs (Fotiadis et al., 2002), or indirectly by modulating the biophysical properties of the IMM lipid bilayer, as demonstrated for other AQPs and cellular organelles (Tong et al., 2012). Hence, it is attractive to speculate that an increase in mitochondrial membrane fluidity by Cd could enhance AQP8 activation.

An additional mechanism for Cd-induced mitochondrial damage due to increased ROS formation may be interference with mitochondrial antioxidative defense enzymes (Figure 1C), leading to decreased expression or reduced functional activity (e.g., by displacement of redox active metal ions from these enzymes, or by complex formation of Cd with sulfhydryl groups). It could involve mitochondrial MnSOD, SOD2, Fe (III)-containing catalase, selenocysteine-containing GPX1 (reviewed in Cuypers et al., 2010), and possibly mitochondrial GSH and glutathione reductase (Ribas et al., 2014).

Cd and Fe damage are compared in Table 1. Strikingly, despite the Fe undergoing Fenton chemistry and Cd not, there is a significant overlap of alterations of mitochondrial structure, function, and expression elicited by increased mitochondrial Fe and Cd, possibly because of displacement of Fe by Cd (see above).

## Therapy

The best and paramount therapy of Fe or Cd nephrotoxicity is its prevention (reviewed in Nawrot et al., 2010; Wish et al., 2018). Apart from symptomatic treatment, there is currently no effective clinical therapy for acute Fe or Cd intoxication. Two treatment approaches found their way in the literature: (1) metal chelating agents without or (2) with antioxidants. The Fe chelating agents currently in use for prevention and management of AKI include deferoxamine, deferasirox and deferiprone, all of which are highly specific for Fe (Sharma and Leaf, 2019). A combination of Fe chelators and antioxidant treatment (e.g., N-acetylcysteine or ascorbic acid) has been proposed for myoglobin-induced AKI (Panizo et al., 2015). Nevertheless, Fe chelators are nephrotoxic, limiting their application (Kang et al., 2019).

Despite detailed knowledge of the *in vitro* molecular properties of chelating agents and antioxidants and their interactions with Cd (see Flora et al., 2008; Bjorklund et al., 2019), their clinical usefulness remains unproven apart from occasional case reports. Succimer, diethylenetriaminepentaacetate, and ethylenediaminetetraacetic acid (EDTA) have been considered as Cd chelating agents for acute Cd intoxication (reviewed in Smith, 2013). In animals, chelators can reduce acute Cd-induced mortality, provided treatment started very soon after Cd ingestion. Zn may exert protective effects against acute Cd toxicity that are partially MT-mediated, as suggested from neonatal murine-engineered cardiac tissues (Yu et al., 2020).

There is no recommended treatment for chronic Cd exposure in humans (reviewed in Smith, 2013), consistent with the Agency for Toxic Substances and Disease Registry (ATSDR) guidelines for Cd (Faroon et al., 2012). In particular, EDTA, was of no benefit in chronic Cd-induced renal dysfunction (Wu et al., 2004). This 14-year follow-up study of 17 patients was important because it challenges case reports suggesting beneficial effects of EDTA applied together with antioxidants, such as GSH (e.g., Gil et al., 2011). No recommended treatment strategy is available for Fe-induced CKD.

## Conclusion and Outlook

Our review has summarized where the science and applications are currently for Fe and Cd entry into renal mitochondria listed in the sections of this review although we had to limit coverage. Still, despite wealth of current knowledge, several burning issues remain unresolved and require clarification in the near future. One hopes, for example, that biophysical methods will reveal Fe concentration and speciation for renal mitochondria. A largely unsolved issue is mitochondrial DMT1 import where several hypotheses concurrently prevail. DMT1 import could originate from the cytosol and involve a (co-translational) import pathway for mitochondrial proteins encoded in the nucleus. The “vesicular” hypothesis implies that DMT1 is targeted to the OMM through transient or permanent interactions with ER or endosomal vesicles.

Additional studies on isolated *b/b* vs. +/+ and +/*b* mitochondria should reveal whether other DMT1 substrates like Cd^2+^ or Cu^1+^ rely mostly on DMT1 to cross the OMM (as for Mn^2+^) or have additional means for entry (like Fe^2+^). The additional means for entry represents a more challenging issue because one must propose and test candidates where the design of the test could depend on the nature of the candidate. Also, finding the extent to which mitochondrial entry via DMT1 and “kiss and run” overlap also requires more hypothesis driven tests. Developing *in vitro* subcellular systems where components can come from different cells as a source could lead to future advances. An additional transport aspect concerns the entry pathways for Fe and Cd at the IMM. Direct proof of Mfrn1- and Mfrn2- dependent Fe transport at the IMM is missing, implying that other yet unknown pathways for Fe (and Cd) may be operative at the IMM as well. Although indirect evidence suggests Cd entry into the matrix via the MCU (Lee et al., 2005b), proof is lacking and would require a combination of molecular biology and electrophysiological approaches, as shown for Ca^2+^ (Kamer and Mootha, 2015; Mammucari et al., 2017).

Concerning the complicated, reciprocal relationship between (patho)physiological ROS and mitochondrial dynamics and plasticity, the role of fusion and fission events in ROS signaling is attracting much attention, postulating compartmentalization of damaging ROS through fission or antioxidant sharing through fusion. Moreover, how does formation of respiratory supercomplexes affect ROS production and balance between superoxide and H_2_O_2_? Changes in membrane lipids influence membrane-associated ROS-producing and ROS-metabolizing systems as well as the ETC and AQPs, therefore the impact of Fe/Cd on membrane lipid alterations affecting mitochondrial health warrants further investigation. Lastly, are mitochondria-derived physiological ROS signals involved in regulating Na^+^/K^+^-ATPase activity, which is indispensable for kidney function?

Fe overload and Cd accumulation occur in both kidney cortex and medulla (reviewed in Thévenod, 2010; Thévenod and Wolff, 2016; Thévenod et al., 2019). But why is Fe and Cd nephrotoxicity less apparent in the distal nephron although transporters for these metal ions, receptors for metal-protein complexes and mitochondria prevail in these nephron segments? The relative resistance of the distal nephron to toxicity by these metal ions may result from its lower sensitivity to oxidative stress, an increased potential for adaptive responses and stress-induced factors (e.g., hypoxia-inducible factor-1α, hepcidin, and NGAL) (reviewed in Thévenod and Wolff, 2016), and its metabolic profile (see section “Nephron Transport and Metabolism: Focusing on Energy”). Indeed, all these issues could also account for the relative resistance of the kidney medulla to damage elicited by other inducers of AKI (Hall et al., 2013). A better understanding of differences in kidney cortex and medulla of transport, metabolism, mitochondrial dynamics, and stress/adaptive signaling may contribute to the development of preventive and novel therapeutic strategies for acute and chronic kidney injury induced by Cd and Fe.

## Author Contributions

FT received the invitation to writing this review who subsequently invited MG and W-KL to join. FT drafted sections “Introduction,” “Nephron Transport and Metabolism: Focusing on Energy,” “Mitochondrial Functions: Determinants of Life and Death,” “Transport at OMM and IMM and Properties in Relation to Metal Ion Movement,” “Fe Overload and Renal Injury,” “Fe and Mitochondrial Damage,” “Cd Toxicity and the Kidney,” “Cd and Kidney Mitochondrial Damage,” “Therapy,” and Figure 4. MG drafted sections “Metal Distribution in Mitochondria – To What Purpose?,” “A Recent, Versatile Candidate for OMM Import of Divalent Cations,” “How Does DMT1 Integrate Into Mitochondrial Metal Homeostasis?,” “Systemic and Cellular Iron Homeostasis,” and Figures 2, 3. W-KL drafted sections “ROS/RNS: Double-Edged Swords,” “Redox Control Mechanisms Through Transport at Mitochondrial Membranes,” and Figure 1. All authors drafted section “Conclusion and Outlook” and reviewed one another’s drafts.

## Conflict of Interest

The authors declare that the research was conducted in the absence of any commercial or financial relationships that could be construed as a potential conflict of interest.
