# Corrigendum: Iron and Cadmium Entry Into Renal Mitochondria: Physiological and Toxicological Implications

**DOI:** 10.3389/fcell.2021.687810

**Published:** 2021-04-19

**Authors:** Frank Thévenod, Wing-Kee Lee, Michael D. Garrick

**Affiliations:** ^1^Faculty of Health, Centre for Biomedical Education and Research, Institute of Physiology, Pathophysiology and Toxicology, Witten/Herdecke University, Witten, Germany; ^2^Department of Biochemistry, Jacobs School of Medicine and Biomedical Sciences, University at Buffalo, Buffalo, NY, United States

**Keywords:** reactive oxygen species, divalent metal transporter 1, ionic mimicry, manganese, copper, nephrotoxicity, acute kidney injury, chronic kidney disease

In the published article, there was an error in the Funding statement for MG. The corrected paragraph appears below:

## Funding

FT received funding from BMBF (01DN16039), DFG (TH345), and ZBAF. MG appreciates the support of grant R01 DK109717 from the National Institute of Diabetes and Digestive and Kidney Diseases and the Office of Dietary Supplements. W-KL received financial support through the Intramural Funding Program at Witten/Herdecke University (IFF 2018-52).

The authors apologize for this error and state that this does not change the scientific conclusions of the article in any way. The original article has been updated.

